# A Method for Quantification of Stretch Reflex Excitability During Ballistic Reaching

**DOI:** 10.1109/TNSRE.2023.3283861

**Published:** 2023-06-16

**Authors:** Thomas A. M. Plaisier, Ana Maria Acosta, Julius P. A. Dewald

**Affiliations:** Department of Biomedical Engineering, Northwestern University, Chicago, IL 60208 USA; Department of Physical Therapy and Human Movement Sciences, Northwestern University, Chicago, IL 60611 USA; Department of Physical Therapy and Human Movement Sciences, Northwestern University, Chicago, IL 60611 USA

**Keywords:** Robotics, stretch reflex, stroke, upper extremity

## Abstract

Stretch reflexes are crucial for performing accurate movements and providing rapid corrections for unpredictable perturbations. Stretch reflexes are modulated by supraspinal structures via corticofugal pathways. Neural activity in these structures is difficult to observe directly, but the characterization of reflex excitability during volitional movement can be used to study how these structures modulate reflexes and how neurological injuries impact this control, such as in spasticity after stroke. We have developed a novel protocol to quantify stretch reflex excitability during ballistic reaching. This novel method was implemented using a custom haptic device (NACT-3D) capable of applying high-velocity (270 °/s) joint perturbations in the plane of the arm while participants performed 3D reaching tasks in a large workspace. We assessed the protocol on four participants with chronic hemiparetic stroke and two control participants. Participants reached ballistically from a near to a far target, with elbow extension perturbations applied in random catch trials. Perturbations were applied before movement, during the early phase of movement, or near peak movement velocity. Preliminary results show that stretch reflexes were elicited in the stroke group in the biceps muscle during reaching, as measured by electromyographic (EMG) activity both before (*pre-motion* phase) and during (*early motion* phase) movement. Reflexive EMG was also seen in the anterior deltoid and pectoralis major in the *pre-motion* phase. In the control group, no reflexive EMG was seen, as expected. This newly developed methodology allows the study of stretch reflex modulation in new ways by combining multijoint movements with haptic environments and high-velocity perturbations.

## I. Introduction

STRETCH reflexes are generally considered in static conditions but play important roles during movement as well, such as stabilizing joints when performing voluntary motions, or correcting for perturbations and sudden changes in limb dynamics. The stretch reflex is a neural response to changes in muscle length that scales with stretch velocity and is formed by a monosynaptic pathway relaying information from muscle spindles to alpha motor neurons in the spinal cord [1], [2], [3], [4]. The magnitude of stretch reflexes depends on motor neuron excitability, Ia afferent presynaptic inhibition at axon terminals, Ia inhibitory interneuron input (i.e. reciprocal inhibition), and the gain of muscle spindles which is controlled by gamma motor neurons [5], [6], [7], [8]. Stretch reflex modulation occurs throughout movement to optimize movement accuracy in the presence of unpredictable perturbations [9]. Only a few research groups have successfully quantified this modulation in the upper limb, due to the difficulty of eliciting the stretch reflex under relaxed conditions [2], [9], [10], [11], [12], and especially during movement [13], [14], [15], [16].

Supraspinal structures modulate stretch reflex excitability based on limb kinematics, muscle activation, and motor task [12], [17]. Direct (noninvasive) measurement of neural activity in these structures with sufficient time resolution is currently not feasible, but stretch reflex responses in muscle activity can be used as a proxy measure of motor neuron excitability and more importantly of supraspinal drive [18], [19], [20]. The study of supraspinal motor drive during functional movements, through measurement of stretch reflex excitability, can further our understanding of the role, expression, and modulation of stretch reflexes during movement, especially in individuals with neurological pathologies. For example, stretch reflex hyperexcitability (also known as spasticity) is a symptom of several central nervous system pathologies including stroke, multiple sclerosis, and cerebral palsy. Upregulation of spinal motor neuron excitability due to disruption of corticospinal and corticobulbar pathways has been proposed as a mechanism for spasticity based on observed stretch reflex behavior [8], [20], [21], [22].

Much of our understanding of the stretch reflex is based on experimental setups that rely on the application of perturbations with various position [17], [23], velocity [22], [24], [25], [26], [27], or torque [1], [12] profiles to single joints, with or without pre-activation of specific muscles [28], [29]. However, the state of the motor system differs greatly between these tasks and the dynamic conditions of volitional movement [5], [14], [30]. For example, supraspinal structures anticipate and account for expected task dynamics and potential disturbances [31], [32] and modulate muscle spindle feedback in preparation for a planned movement [33]. This emphasizes the need for studying reflex behavior during voluntary movement, which requires devices capable of eliciting and measuring stretch reflexes under dynamic conditions. To this end, we developed a new methodology and robotic platform for the quantification of stretch reflex responses in the upper limb during volitional, multijoint movements. Reflexes are elicited by applying ramp-and-hold angular velocity perturbations aimed at perturbing the arm (single or multijoint) while participants perform a reaching task.

Previous works have used multiple methods to elicit and measure the stretch reflex including 1) self-generated limb movement, 2) peripheral nerve stimulation (to elicit Hoffman or H-reflexes), or 3) mechanical perturbations. Self-generated limb movement tasks have been used in participants with chronic hemiparetic stroke and healthy individuals, relying on participants ballistically moving a joint through its range of motion in the absence of perturbations and other external influences. However, the movement velocities achieved in the presence of abnormal muscle synergies, often seen following a stroke, are generally insufficient to elicit stretch reflexes [14], [34], [35]. H-reflexes measured during various dynamic tasks like walking and stepping with the lower limb [36], [37], [38], [39] have been used to show active, task-specific modulation of stretch reflex excitability in healthy individuals in contrast to the absence of modulation in spastic stroke. However, this method cannot be generalized to all muscles and joints, because H-reflexes can only feasibly be elicited by stimulating the tibial nerve or median nerve [40], [41]. Additionally, H-reflexes bypass muscle spindles which play an important role in providing sensory feedback of muscle length and change in length (i.e. stretch velocity) to the nervous system [40]. Mechanical perturbations offer myriad ways to elicit stretch reflex responses and measure system dynamics [16], [23], [24], [25], [26], [27], [29], [42], [43]. Ramp-and-hold perturbations have been used successfully to elicit stretch reflexes, primarily in single joints like the elbow and ankle [23], [43], [44]. An advantage of these mechanical perturbations is that they allow for consistent kinematic input across individuals (e.g. joint angular velocity during the perturbation) regardless of volitional movement velocity.

In summary, studies examining the stretch reflex in humans have thus far largely focused on nonvolitional motor tasks and single joints, foregoing the effect of descending motor drive during active movement and reflex coordination between joints. In our proposed method, we apply angular velocity perturbations that combine rapid accelerations with constant angular velocity plateaus sufficient to elicit the stretch reflex at precise points during movement. This new method and robotic platform allow us to combine multijoint movements of the upper limb with the high-velocity perturbations needed to quantify stretch reflex modulation during volitional movement, while simultaneously simulating haptic environments by for example adding shoulder abduction loads while reaching.

## II. Methods

### A. Participants

4 participants with chronic hemiparetic stroke (1 female, 3 male) and 2 control participants (both male) participated in the study. Stroke participants who were able to generate some reaching movement without external support of the arm and were able to actively raise the arm to 80° abduction (90° being horizontal) were included in the study. Only participants with chronic stroke (> 1 year post stroke) were included to avoid the effects of subacute recovery on motor function. The severity of stroke participants’ upper limb motor impairment was 35 ± 17 (mean ± SD) out of 66 as quantified by the upper extremity portion of the Fügl-Meyer Motor Assessment (FMA) scale. Participants were not included/excluded based on FMA. Control participants were included if they had no neurological or physical impairments affecting movement control of the upper limb. Fewer participants were recruited for the control group since they were not expected to show short-latency reflexive responses under the tested perturbation conditions and stretch velocities [22]. The experimental protocol was approved by the Institutional Review Board of Northwestern University, Evanston, IL, and informed consent was obtained prior to participation.

### B. Setup

**Fig. 1.**
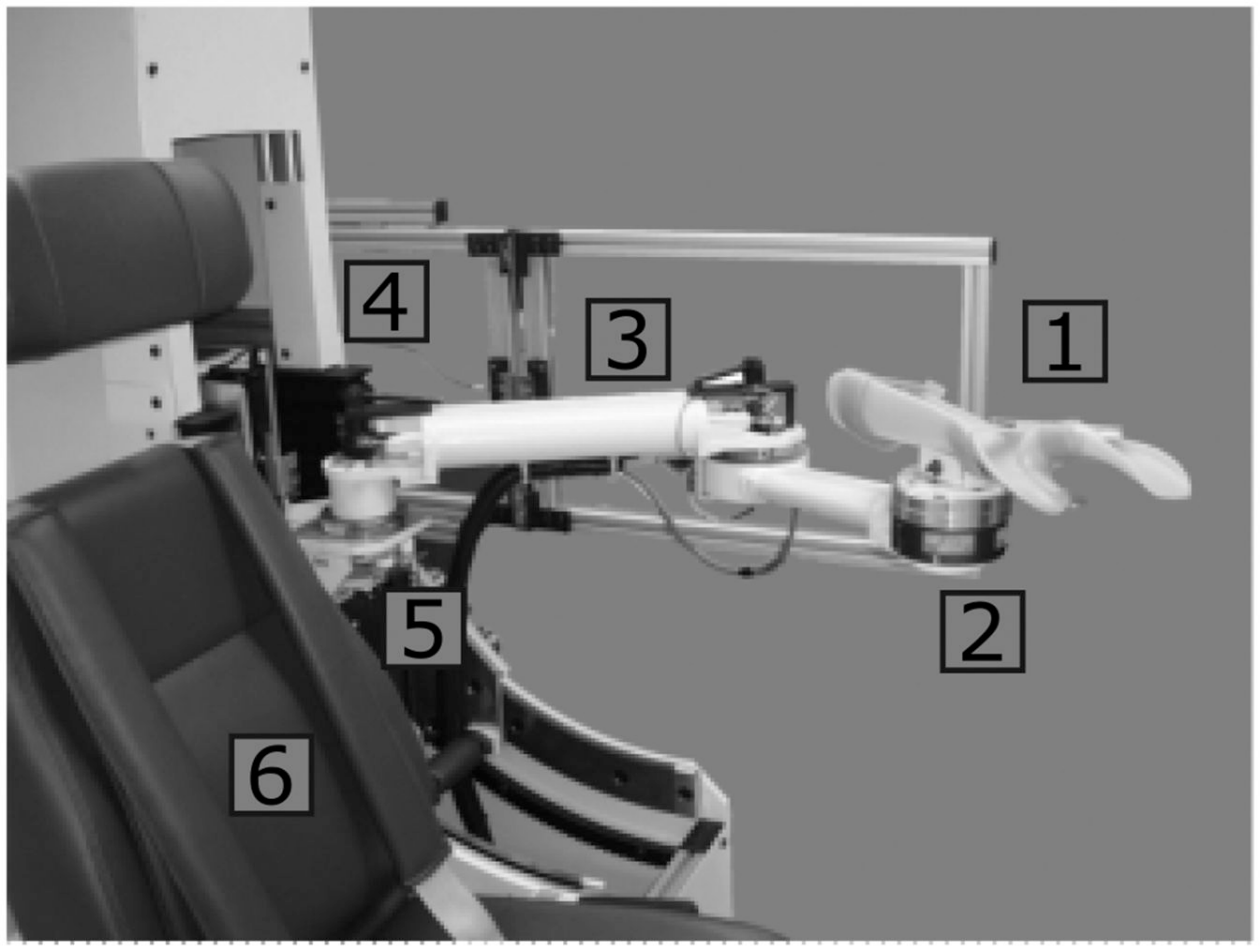
Overview of the NACT-3D, shown here in a left-handed configuration. The robot implements a newly developed haptics platform capable of high-velocity perturbations of 270 °/s at the elbow during volitional motion. Components: 1) Orthosis to couple participant forearm to NACT-3D. 2) 6 DOF force/torque sensor for admittance controller input. 3) Two-link arm with encoders and quick-release mechanism for changing left-/right-handed configuration. 4) Housing of the NACT-3D with operating computer and servos. 5) Vertical platform and work plane adjustment mechanism. 6) Biodex chair.

Participants were seated in a Biodex System 4 chair (Biodex Medical Systems, Inc., Shirley, NY), with the arm coupled to the NACT-3D via a forearm orthosis (see Fig. 1). The NACT-3D, short for New Arm Coordination Training in 3D, is a custom-built two-link planar robot with 3 degrees-of-freedom (DOF): two for movement on the plane, and one for movement perpendicular to the plane. Additionally, the orientation of the plane relative to horizontal can be adjusted. The NACT-3D is instrumented with a 6 DOF force/torque sensor (45E, JR3 Technologies, Inc., Woodland, CA) on the endpoint and encoders to track the proximal and distal links and the orientation of the participant’s forearm. The NACT-3D can be configured to operate as a haptic device driven by forces applied to the endpoint by the participant (admittance controller), or as a traditional robotic device driven by velocity commands (position controller), to follow endpoint trajectories specified by the experimenter. The switch between these two modes is seamless to the participant and allows the application of angular velocity perturbations to the participant’s arm while performing voluntary movements. Participant joint kinematics were calculated from inverse kinematics based on the robot endpoint position, orientation of the interface orthosis, and participant-specific parameters including segment lengths and the position of the shoulder joint relative to the NACT-3D (which was assumed to be constant); see section IV and Fig. 1.

Muscle activity from the biceps brachii (BIC), triceps brachii (TRI), horizontal fibers of the pectoralis major (PEC), and the anterior, posterior, and medial parts of the deltoid (A/P/MDL) was recorded using an 8-channel differential EMG setup (Delsys by Bagnoli System, Inc., Natick, MA). Endpoint forces, EMG data, and calculated endpoint position and participant kinematics were recorded with a sampling rate of 1 kHz and analyzed offline using custom software written in MATLAB (Mathworks, Inc., Natick, MA).

**Fig. 2.**
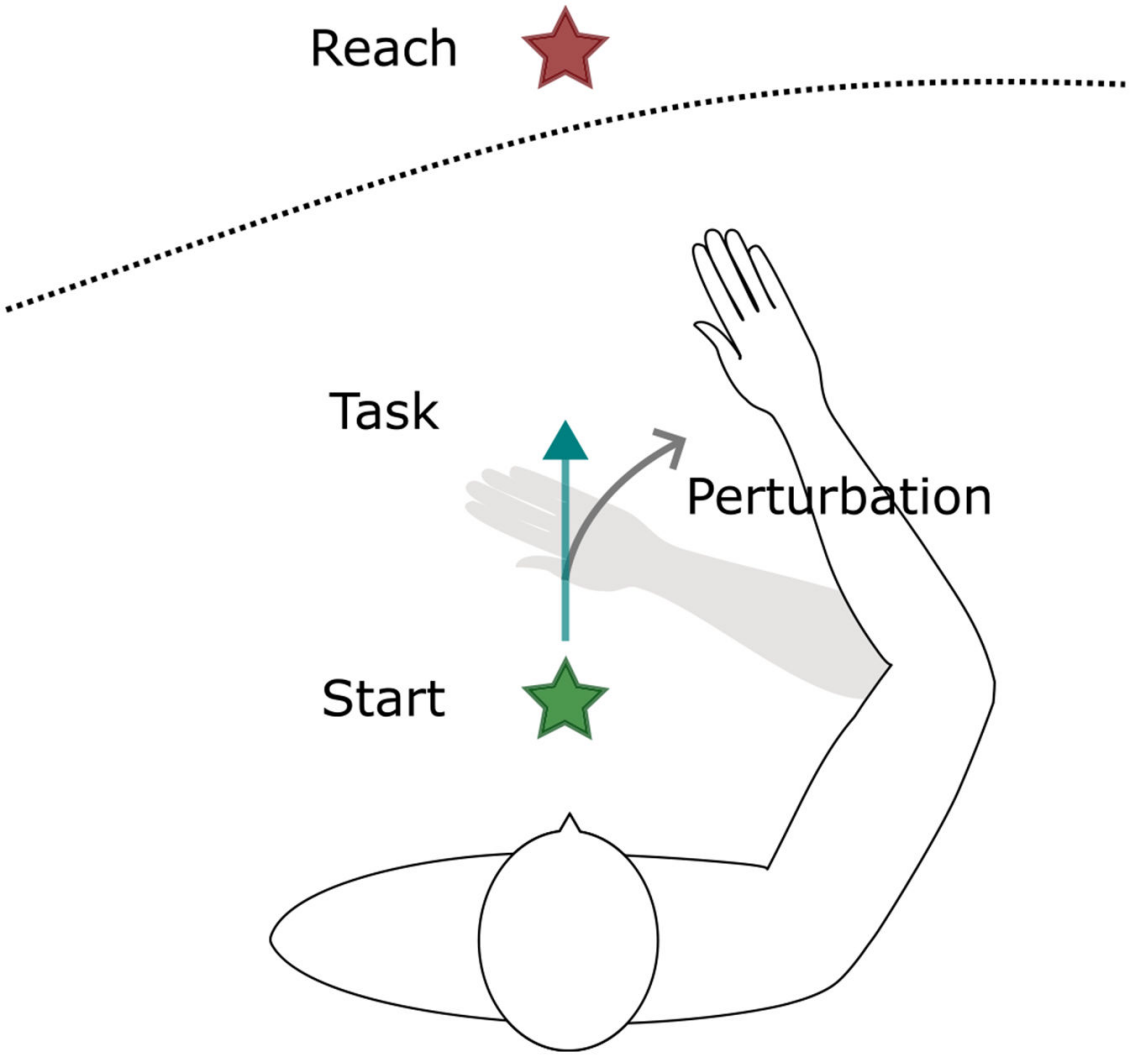
Overview of the task. The participant is seated and connected to the NACT-3D (not shown) via an orthosis. The start target (green star) is placed 10 cm in front of the sternum, while the reaching target (red star) is placed 5 cm beyond the participant’s maximal reaching distance (black dotted line) also in front of the sternum. During the task, the participant is asked to move ballistically from the start target to the reaching target as indicated by the teal arrow. During perturbations (gray arrow), which occur randomly before movement initiation, early during the movement, or near peak movement velocity, the NACT-3D rapidly extends the elbow to elicit a stretch reflex in elbow flexor muscles.

Chest movement and shoulder translation were constrained with straps to ensure that the task and perturbation only resulted in elbow and/or shoulder rotation on the plane of movement. The upper limb was coupled to the robot’s endpoint through an orthosis, with the arm elevated to 80° shoulder abduction. A monitor displaying an avatar of the participant’s trunk, limb, and hand was placed in front of the participant. The monitor also provided visual feedback of start and reaching targets; see Fig. 2. The two targets were placed in a horizontal plane corresponding to 80° shoulder abduction, with the start target 10 cm in front of the sternum and the reaching target 5 cm beyond the participant’s maximal reaching distance along the sagittal plane, to encourage maximal reaching effort. The maximal work area of the participant while moving on the virtual table was obtained and used to set outer boundaries for the movement of the NACT-3D endpoint for safety. Additional safety precautions included a velocity limit equivalent to a 300 °/s perturbation for the endpoint, and force limits for each servo motor. Participants performed the protocol first with their dominant or nonparetic limb to attain familiarity with the task, followed by the nondominant or paretic limb.

### C. Perturbation

Elbow (flexion/extension) and shoulder (flexion/extension) joint kinematics of the participant’s arm were estimated in real-time based on the NACT-3D endpoint position and the shoulder position relative to the NACT-3D. The shoulder position was calculated during setup in a standardized position (90° elbow flexion and 45° shoulder flexion) and assumed to be constant throughout the experiment. To apply the perturbation, the NACT-3D controller was switched from haptic mode (admittance control) to position control mode where the endpoint was moved along a circular trajectory perpendicular to the participant’s forearm and centered at the participant’s elbow (see Fig. 2). The perturbations followed a ramp-and-hold angular velocity profile with a ramp-up time of 20 ms to a plateau angular velocity of 270 °/s which was maintained for 30 ms. The desired endpoint trajectory and angular velocity were updated at a rate of 1 kHz to account for movement of the limb. Following the perturbation plateau, the velocity of the endpoint was returned to its value at perturbation onset over the course of 20 ms, and the controller was switched back to admittance control.

### D. Task

**Fig. 3.**
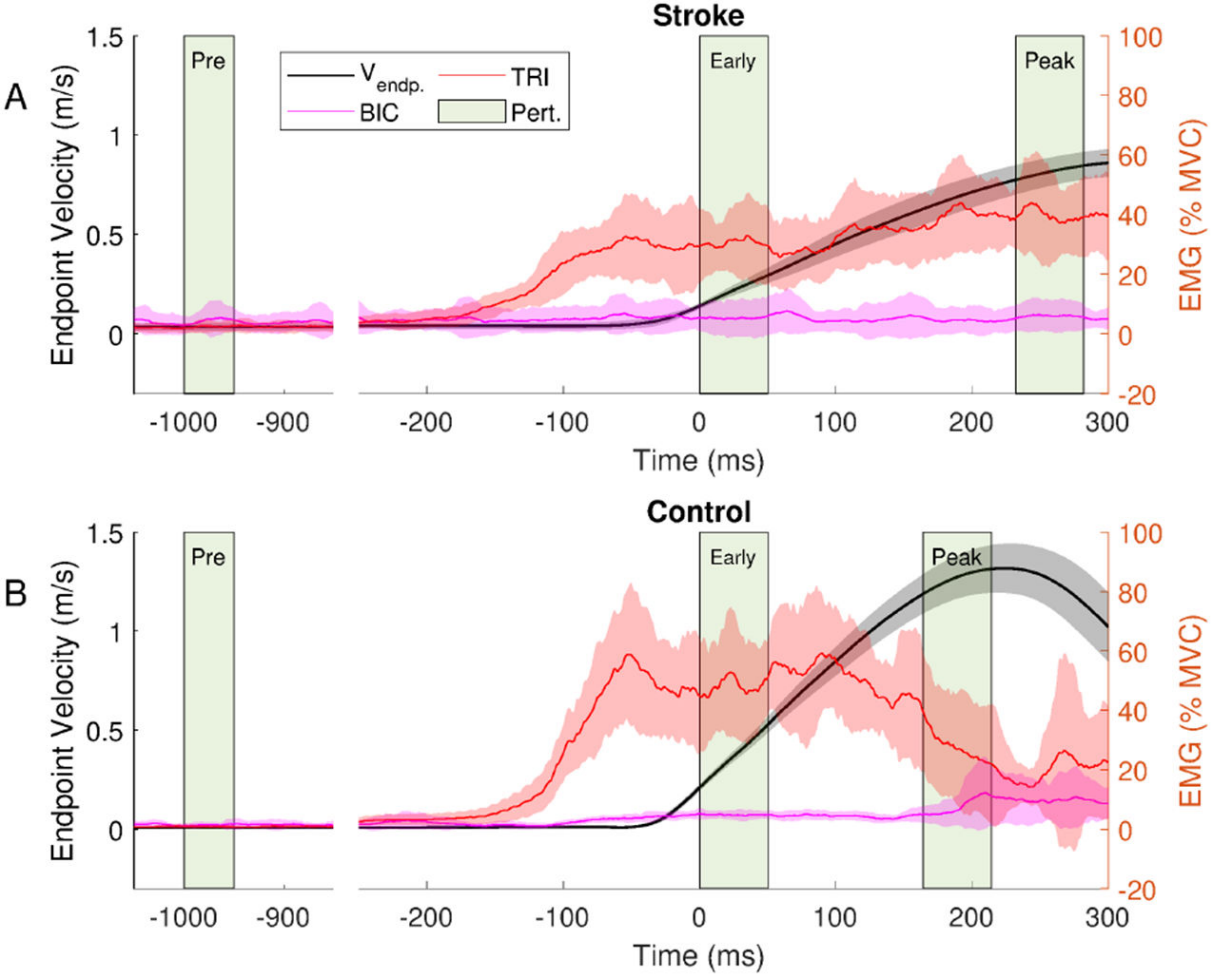
Representative traces from a stroke (top panel) and a control (bottom panel) participant during unperturbed reaching trials to show the three movement phases. Endpoint velocity is shown on the left axis, and normalized EMG of the biceps (BIC) and triceps (TRI) muscles are shown on the right axis. Trials are aligned to the start of the *early motion* phase (time 0 ms). The shaded blocks indicate the starting point and time windows of perturbations for all three motion phases (from left to right *pre-motion* at 1 second prior to expected movement start, *early motion* at 15% MVV, and *peak motion* at 90% MVV). In catch trials, perturbations are applied at the start of one of these motion phases. Traces show the mean (solid lines) ± 1SD (shaded areas) across trials of the nondominant (control) or paretic (stroke) limb. The stroke participant’s TRI EMG continues past the elbow extension velocity peak, with minimal BIC EMG activity. In contrast, in the control participant movement was preceded by an increase in triceps activity followed by a decrease in triceps and increase in biceps activity near peak movement velocity.

Participants were asked to perform a series of reaching movements on a virtual frictionless haptic table during three trials to measure their Maximal Voluntary Velocity (MVV), used to normalize the timing of the perturbation in each motion condition. Participants were asked to reach as fast as possible from the start target to the reaching target, yielding velocity curves similar to those shown in Fig. 3. Peak endpoint velocity (defined as the magnitude of the endpoint velocity vector in three dimensions) was extracted, and MVV was calculated as the average peak velocity across the three trials.

Maximal Voluntary Contractions (MVC), used to normalize EMG measurements within muscles, were collected in separate isometric trials using the same device [45]. Participants were asked to generate an MVC with the joint and in the direction that maximized muscle contraction for each muscle being measured, holding the contraction for 3 s in three consecutive trials with 20 s of rest in between. The average rectified EMG over a window of 500 ms centered at the peak was extracted for each trial and averaged across the three trials.

Participants were asked to perform ballistic reaching motions from the start target to the reaching target with a 3 s rest before movement to determine baseline activity. Participants were encouraged to reach for the target as fast as possible and prioritize high reaching velocity over accuracy. During catch trials, the device applied perturbations to extend the elbow joint randomly at one of three phases of the reaching motion: 1) *pre-motion* (2 s after obtaining the home target and before movement initiation), 2) *early motion* (at 15% MVV in the acceleration phase), and 3) *peak motion* (at 90% MVV in the acceleration phase). Participants were instructed to not react to the perturbation and to relax after the perturbation occurred. Fatigue was monitored through peak velocity in each trial and regular rest breaks were provided. Each perturbation trial was followed by a series of one to five unperturbed trials with the number determined randomly. This was repeated until participants completed three perturbation trials for each motion phase (in this group 39 ± 6 unperturbed trials were completed per participant).

### E. Data Analysis

The collected data was cleaned based on the endpoint kinematics to ensure that all perturbed trials had a peak velocity > 90% MVV, and that the endpoint velocity followed the desired trajectory.

EMG data was bandpass filtered by the Delsys system at 20-450 Hz before digitization, then digitally filtered with a 60 Hz, 6th order, zero-phase, elliptical notch filter to reject mains noise, detrended (1st order), rectified, smoothed with a zero-phase 10 ms moving average window, and normalized to 100% MVC.

Reflexive EMG for each motion phase was quantified in the short-latency reflex (SLR, 25-75 ms) window, and long-latency reflex (LLR, 75-125 ms) window, following the perturbation [3], [19], [22].

Reflexive EMG was calculated as the difference between the average processed EMG across the SLR or LLR window in perturbed trials and the average processed EMG during the equivalent time window (based on % MVV) of all unperturbed trials for a given motion phase. Positive reflexive EMG values indicate an increase in muscle activity in the SLR or LLR window in response to the perturbation compared to unperturbed trials, while negative reflexive EMG values indicate a decrease in muscle activity.

## III. Preliminary Results

Fig. 3 illustrates elbow and shoulder joint velocity, and BIC and TRI muscle activity during typical reaching behavior for a representative stroke (top panel) and control (bottom panel) participant. Both velocity and muscle activity traces were ensemble averaged across trials, and traces were aligned to the start of the *early motion* phase (time 0 ms). SLR windows for all three motion phases (*pre*-, *early*, and *peak motion*) are indicated. Note that the reaching movement to the target mainly required elbow extension and shoulder flexion, reflected by muscle activation in the TRI, ADL, and PEC muscles (of which only TRI is shown).

MVV in the stroke group was 0.78 ± 0.24 m/s, and 1.09 ± 0.17 m/s in the control group. Participants were able to reach peak velocities of at least 90% MVV during the task in 94% of all trials; trials with lower peak velocity were discarded. TRI activity preceded movement onset by 140 ± 98 ms in stroke and 110 ± 28 ms in control. The muscle activation profile of TRI and BIC in the control group showed the triphasic pattern [46] of a rapid increase in extensor EMG before acceleration at the start of the movement, followed by a 200 ms plateau and a gradual decrease near peak velocity. BIC activation was constant throughout the initial movement phases and increased after reaching peak velocity, consistent with a braking action after reaching the target [45]. In contrast, in the stroke group, TRI muscle activity was sustained for several hundred milliseconds, in some cases even beyond task completion. Background BIC activity during relaxed/quiescent periods was quantified at the start of the *pre-motion* phase in unperturbed trials by comparing average EMG across a 100 ms window to zero. BIC background activity was seen in both cohorts (3.9 ± 2.4% MVC for stroke, 1.6 ± 1.2% MVC for control).

**Fig. 4.**
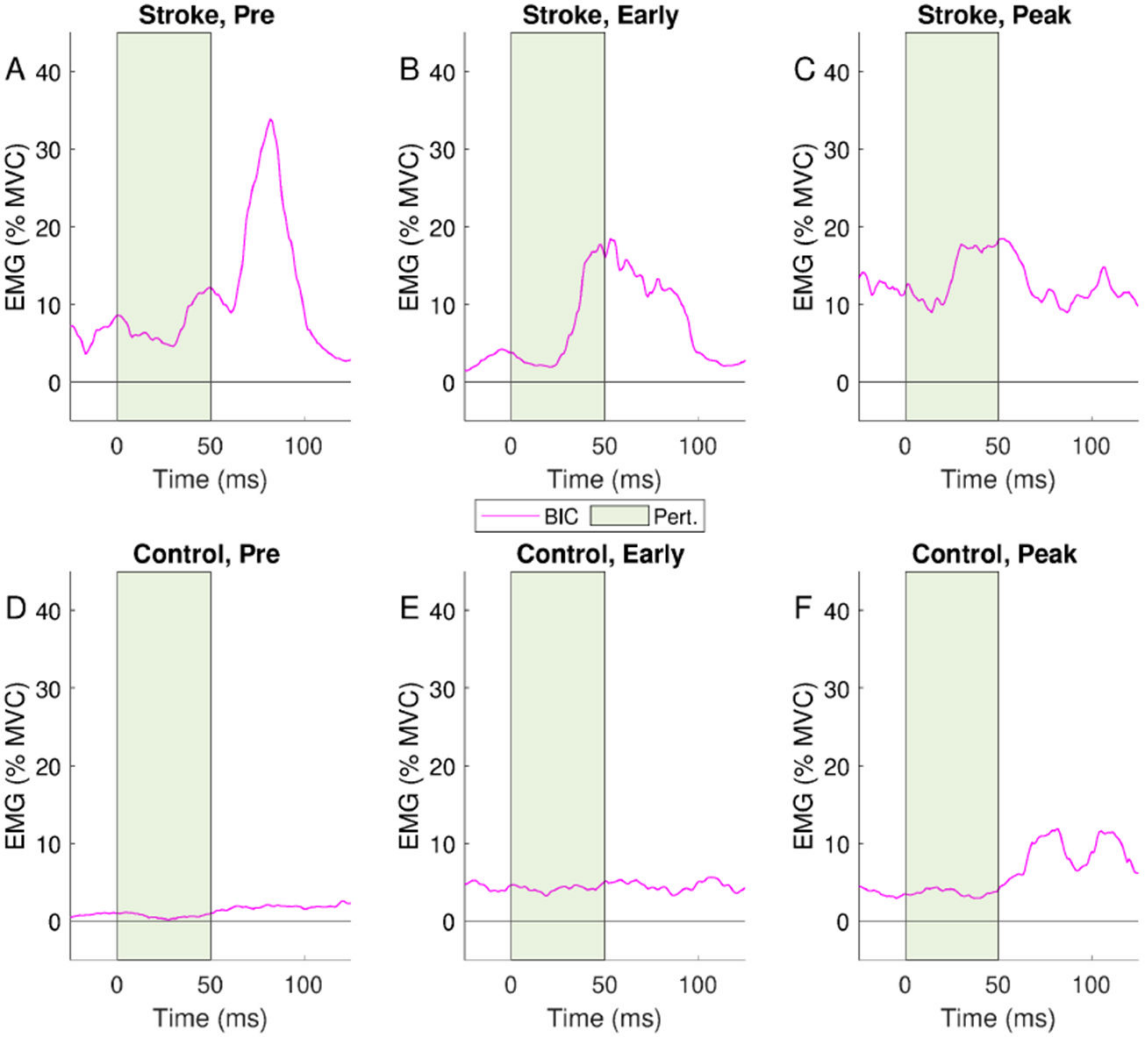
Representative traces from a stroke (top row) and a control (bottom row) participant during perturbed trials, showing average biceps (BIC) EMG normalized to % MVC. Trials are aligned to the start of the perturbation at time 0 ms, with the perturbation indicated by the green shaded block. An increase in BIC EMG in the SLR window (time 25-75 ms) can be seen in the traces of the stroke participant, especially following the *early* and *peak motion* perturbations. EMG in the LLR window (75-125 ms) is greatest during the *pre-motion* phase. The control participant shows no increase in EMG during the SLR window in the *pre*- and *early motion* phase, with a small increase during the LLR window during *peak motion*.

Fig. 4 shows the BIC EMG response to the perturbation applied at each phase for representative stroke (top row) and control (bottom row) participants. In the stroke participant, reflexive activity is visible as an increase in EMG starting as early as 25 ms after perturbation onset and lasting for the duration of the SLR window. An increase in BIC EMG was also seen starting around 75 ms following *pre-motion* perturbations for the stroke participant and after *peak motion* perturbations for the control participant; these time delays are indicative of long-latency reflexes. The control participant showed no discernible reflexive responses during the SLR window to perturbations in the pre- and *early motion* phases of movement. Similar responses were seen in other participants’ trials where reflexes were elicited.

**Fig. 5.**
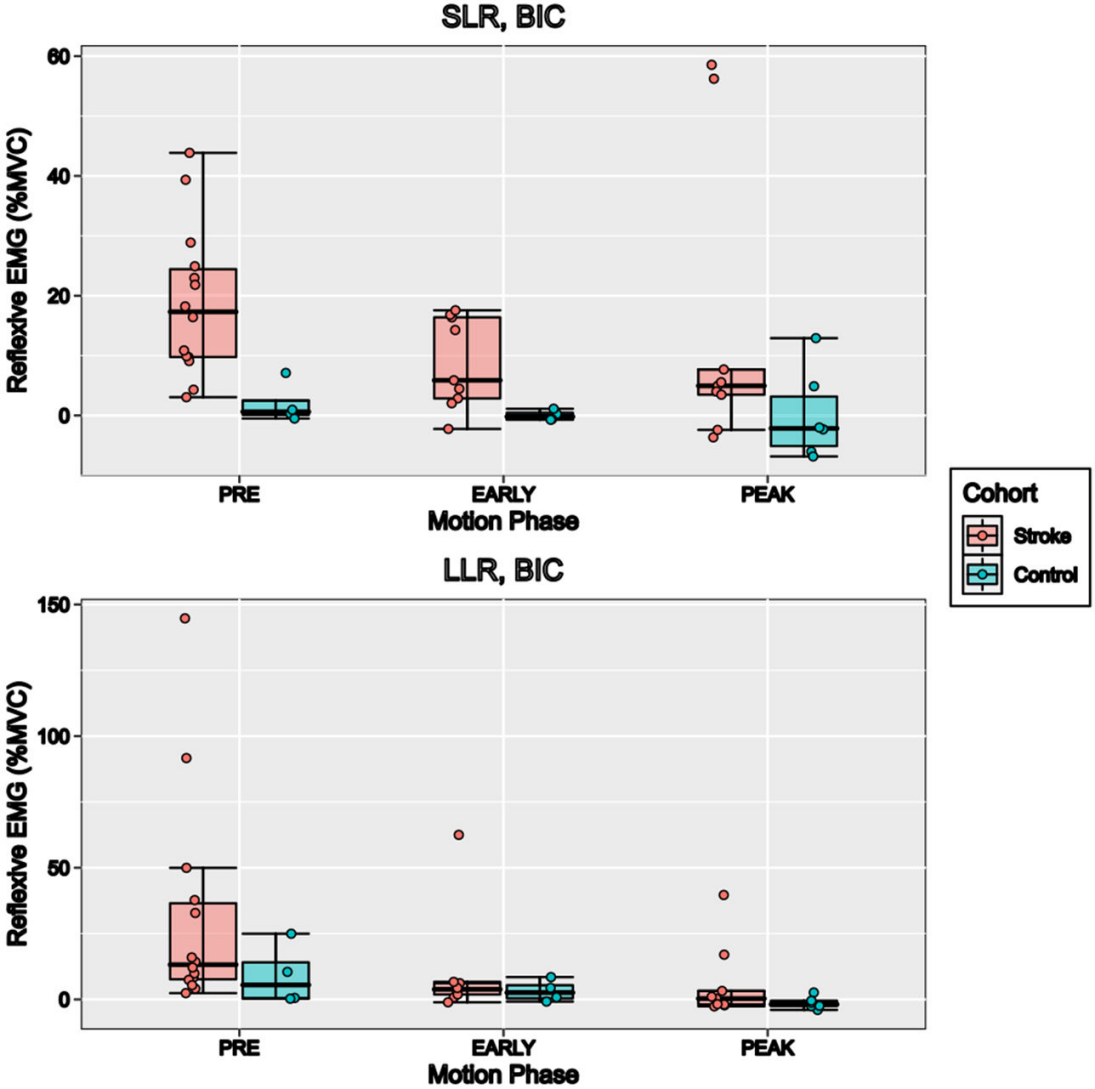
Average reflexive BIC EMG across all participants and motion phases, normalized to % MVC. Reflexive EMG was defined as the difference between average EMG for a given time window following perturbations, and the average of the equivalent time points following the start of each motion phase in the unperturbed trials. Positive reflexive EMG values indicate an increase in muscle activity following the perturbation compared to unperturbed trials, while negative reflexive EMG values indicate a decrease in muscle activity. Shown are the short-latency reflex window (SLR, top panel) or long-latency reflex window (LLR, bottom panel), with stroke indicated in red and control indicated in blue. Participants in the stroke group show high reflexive EMG in the SLR window, with a trend of decreasing reflexive activity from *pre*- to *peak motion*. Control participants show consistently low reflexive muscle activity across all motion phases.

Fig. 5 shows the group-average reflexive EMG of the BIC SLR (top panel) and LLR (bottom panel) windows following perturbations in the three motion phases. In the stroke group, SLR reflexive BIC EMG was seen for the pre- and *early motion* phases (17.5% ± 13.2% MVC, and 8.3% ± 7.8% MVC, respectively). Reflexive SLR EMG (16.4% ± 12.5% MVC) and LLR EMG (35.1% ± 23.6% MVC) were also found in the PEC muscle of the stroke group during the *pre-motion* phase. In the control participants, no SLR or LLR BIC EMG was seen across all phases.

## IV. Discussion

We present a new methodology for measuring stretch reflex excitability in the upper limb during movement, by applying elbow extension ramp-and-hold velocity perturbations with a novel custom haptic robotic platform. This is done at various points before and during a ballistic reaching task while quantifying muscle activity with surface EMG. We elicited reflexive muscle activity in the biceps muscle both before motion and during motion in the stroke group, but not in the control participants, as was expected based from reciprocal inhibition due to triceps activity during the task. These results present an important step in quantifying stretch reflexes during multijoint movement, which is more reflective of functional tasks than often-studied static conditions.

Following a stroke, the perturbation velocity required to elicit stretch reflexes (threshold velocity) is decreased, due to involuntary background muscle activation or tone (inability to fully relax muscles), increased motor neuron excitability due to monoaminergic influences, and less effective reciprocal inhibition of flexors during a forward reach [20], [47], [48]. Although stretch reflexes can be elicited at rest in individuals without neurological injuries, they require stretch velocities greater than those used in this study [49], or muscle pre-activation to decrease the threshold velocity [22], [50]. The goal of this study was to apply our novel method to quantify stretch reflexes during reaching tasks reflective of activities of daily living and thus precluded pre-activation of the BIC muscle, explaining the absence of stretch reflexes in the control participants.

It should be noted that the perturbations used in our novel method also elicited reflexive muscle activity in shoulder muscles, possibly due to a slight misalignment of the direction of the perturbation with the orientation of the forearm. An important advantage of the robot-based method presented here is the ability to allow multijoint movement and apply controlled perturbations in the desired direction on the plane of movement, thus allowing future characterization of the stretch reflex in multiple muscles acting at the elbow and/or shoulder joints by changing the direction of the perturbation. Future work will also explore the role of the shoulder by applying shoulder flexion/extension perturbations and comparing this with reflexive activity in response to only elbow perturbations.

The results presented here apply to elbow extension perturbations during reaching, thus requiring primarily triceps activation to extend the elbow. In this pilot study, we used a ballistic reaching task to standardize neural drive across the two cohorts. As a result, triceps activity during the task was high which would typically cause reciprocal inhibition of the biceps, effectively modulating stretch reflex excitability. The fact that our results show reflexive biceps activity during the *early motion* phase in stroke but not in control suggests reduced reciprocal inhibition in stroke. The setup can also be used to investigate other neural mechanisms involved in motor control in participants both with and without neurological injuries, such as the influence of background activation (e.g. by introducing a bias force or torque), muscle synergies (by modulating shoulder abduction loading), and the effects of pharmacological drug probes on motor neuron and associated reflex excitability during ballistic and self-paced movement [50].

The newly developed method was evaluated on a small group of stroke participants with a broad range of impairment levels to show its potential in eliciting reflexes. We show that we can use the method to elicit short-latency reflexive activity in participants across this wide range of impairment levels and provide preliminary data demonstrating the potential of our approach. Future work with a larger group size and predefined ranges of impairment levels will be necessary to allow for differentiation between participants with severe spasticity compared to those with mild or no spasticity and allow for a more in-depth investigation of reflex excitability and the underlying neural control.

In conclusion, we have developed a new methodology for quantifying stretch reflex excitability during upper limb movement. In the preliminary data presented here, stretch reflexes of the elbow muscles were elicited by applying an elbow extension perturbation while participants performed a reaching task to a target in front of their torso. Preliminary results of the short-latency reflex indicate that stretch reflexes were elicited in the biceps in the stroke group both while stationary and during the reaching motion. In the control group, stretch reflexes were absent, due to reduced or absent biceps activity at the time of the perturbation and/or insufficient stretch velocities. Preliminary results for the long-latency reflex show significant reflexive activity in the biceps and pectoralis major of stroke participants prior to motion, and higher variability across other muscles compared to the short-latency time window. In addition to the perturbations of the elbow shown here, we can use the method to apply perturbations to the shoulder during reaching and to examine stretch reflexes at the elbow and shoulder during active shoulder abduction as is required in activities of daily living. The proposed methodology opens up new ways of investigating the coordination of stretch reflexes between joints of the upper limb during functional movement tasks.

## Appendix A - Participant Arm Inverse Kinematics

Participant arm inverse kinematics are estimated online based on the endpoint position of the NACT-3D and the measured angle of the orthosis with the robot’s manipulator. During setup, several parameters are measured and entered into the system: segment length of the participant’s forearm between elbow and orthosis, segment length of the upper arm (humerus), and the position of the shoulder rotational joint in the robot’s coordinate frame. The shoulder is kept in place with straps to minimize movement, and its position is assumed to be constant relative to the robot.

Here the following variables are used:

$P_{pe}$ is the position of the participant’s elbow.

$P_{r}$ is the position of the robot’s endpoint.

$P_{ps}$ is the position of the participant’s shoulder (glenohumeral joint).

$\theta_{O}$ is the angle of the orthosis in the global coordinate frame.

$\theta_{ps}$ is the angle of the participant’s upper arm segment relative to the global coordinate frame.

$\theta_{pe}$ is the angle of the participant’s forearm segment relative to the upper arm segment.

$L_{sf}$ is the length of the participant’s forearm segment.

Subscripts x, y, and z indicate Cartesian components within these positions.

The elbow position is estimated based on the orthosis angle and segment lengths through geometry:

$$P_{pe}=P_{r}-\left[\begin{array}{c} \;cos(\theta_{O})\cdot L_{sf}\\ \;sin(\theta_{O})\cdot L_{sf}\\ 0 \end{array}\right]$$

The participant’s shoulder angle is based on the position of the elbow and shoulder:

$$\theta_{ps}=\text{arctan}(P_{pe_{y}}-P_{ps_{y}},\;P_{pe_{x}}-P_{ps_{x}})$$

The participant’s elbow angle is based on the position of the robot’s endpoint and the participant’s elbow.

$$\theta_{pe}=\text{arctan}(P_{r_{y}}-P_{pe_{y}},\;P_{r_{x}}-P_{pe_{x}})-\theta_{ps}$$
